# A case of acute myocardial infarction due to cardiovascular syphilis with aortic regurgitation and bilateral coronary ostial stenosis

**DOI:** 10.1186/s40792-016-0267-x

**Published:** 2016-11-22

**Authors:** Mutsuo Tanaka, Minoru Okamoto, Toshihiko Murayama

**Affiliations:** 1 Department of Cardiovascular Surgery, National Hospital Organization Kumamoto Medical Center, 1-5 Ninomaru, Chuou-ku, Kumamoto, 860-0008 Japan; 2 Department of Pathology, National Hospital Organization Kumamoto Medical Center, 1-5 Ninomaru, Chuou-Kumamoto, 860-0008 Japan

**Keywords:** Syphilis, Acute myocardial infarction, Coronary artery bypass grafting, Aortic valve replacement

## Abstract

We report an interesting case of a 66-year-old man with acute myocardial infarction (AMI) with bilateral coronary ostial stenosis cardiovascular syphilis complicated by aortic regurgitation (AR). A 12-lead electrocardiogram and blood tests on arrival suggested AMI, and echocardiography showed moderate AR. Emergency coronary angiography showed bilateral coronary ostial stenosis. The patient underwent emergency surgical treatment, coronary artery bypass grafting, and aortic valve replacement with a bioprosthetic valve. On arrival, rapid plasma reagin and *Treponema pallidum* hemagglutination tests were 172.2- and 1187.5-fold, respectively. These results suggested cardiovascular syphilis, which was confirmed by pathological findings. The postoperative course was uneventful and the patient was transferred to another hospital on postoperative day 25. This patient received intravenous penicillin for 2 weeks and subsequently oral amoxicillin. When both AR and coronary ostial stenosis are found, it is necessary to consider the presence of cardiovascular syphilis.

## Background

Syphilis is an ancient disease and an important issue internationally among sexually transmitted diseases [1]. Cardiovascular syphilis (CVS) is classified as the tertiary stage of syphilis infection, and it occurs 20–30 years after the initial infection in about 10% of untreated patients [2]. Many reports have shown an association with aortic aneurysm [3] and coronary ostial stenosis [4]. However, CVS has not been encountered recently in Japan. Here, we report a patient with acute myocardial infarction (AMI) and CVS, which were associated with both aortic regurgitation (AR) and bilateral coronary ostial stenosis.

## Case presentation

**Fig. 1:**
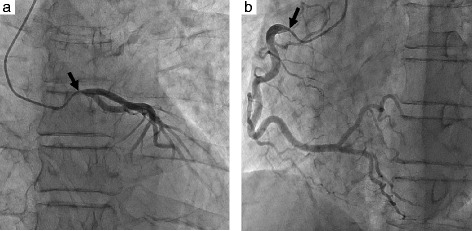
Preoperative coronary angiography. **a** Left coronary artery. **b** Right coronary artery. The stenotic lesions can be seen in bilateral coronary ostia (*arrow*)

The patient was a 66-year-old man who presented with continuous and worsened chest pain. A 12-lead electrocardiogram, recorded on admission, demonstrated ST elevation in leads V1-4, aVR, and ST as well as depression in II, III, aVF, and I. Transthoracic echocardiography (TTE) showed moderate AR and diffuse severe hypokinesis of the left ventricular wall. Laboratory findings showed that the cardiac enzymes, creatine kinase and creatine kinase-MB, were slightly elevated at 287 and 42 IU/l, respectively. Furthermore, rapid plasma reagin (RPR) card and *Treponema pallidum* hemagglutination (TPHA) tests were 172.2- and 1187.5-fold, respectively. Subsequently, emergency coronary angiography was performed and revealed bilateral coronary ostial stenosis (Fig. 1). Emergency surgical treatment with aortic valve replacement and coronary artery bypass grafting (CABG) was planned; preoperative intra-aortic bloom pumping (IABP) prior to surgery was avoided because of AR.

**Fig. 2:**
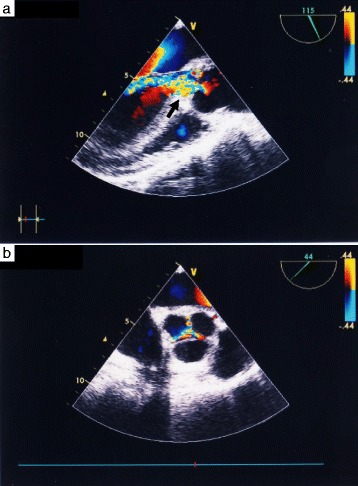
Preoperative transesophageal echocardiography (just after the induction of systemic anesthesia). **a** Moderate and wide aortic regurgitation can be seen (*arrow*). **b** The shape of the aortic valve was tricuspid

**Fig. 3:**
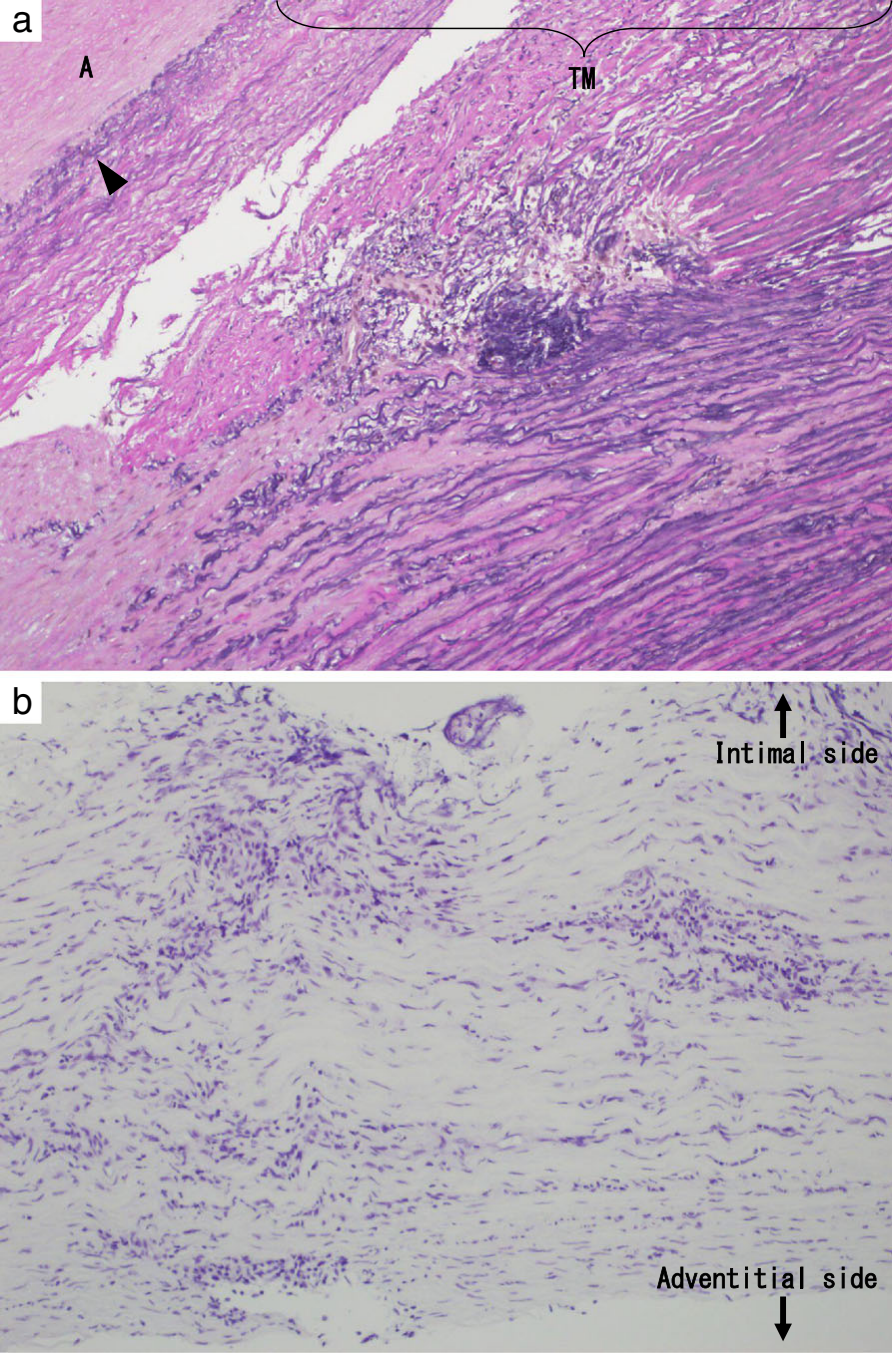
The pathological images of the aortic wall and aortic valve. **a** The pathological image of aortic wall. The infiltration of inflammatory cells composed mainly of neutrophils and lymphocytes localized in the tunica media, and it is accompanied by angiogenesis. Cystic medial necrosis findings are not detected. *A* atheroma, *TM* tunica media, *arrow head*, internal elastic lamina. **b** The pathological image of aortic valve. Myxoid changes, fibrosis, and the partial infiltration of inflammatory cells were detected. Hematoxylin and eosin-stained section, magnification ×100 (**a**, **b**)

Transesophageal echocardiography (TEE) just after the induction of systemic anesthesia revealed moderate AR as in the preoperative TTE (Fig. 2). Surgery was performed via a median sternotomy; the left internal thoracic artery (LITA) and great saphenous vein were harvested in situ for the bypass graft. Cardiopulmonary bypass (CPB) was established with ascending aorta (Asc-Ao) and bilateral vena cava cannulations, and three stay sutures were placed on the posterior side of the pericardium to enable visualization of the coronary artery. At first, CABG to the right coronary artery with the saphenous vein graft (SVG) was performed on heart-beating, then CABG to the left circumflex artery (LCx) was attempted, but it failed because ventricular fibrillation and left ventricular (LV) expansion occurred regardless of the addition of the LV vent tube via the right upper pulmonary vein. Adhesion of the Asc-Ao with the surrounding fat tissue was found on maneuvering the Asc-Ao, and aortic wall thickening and narrowing of the bilateral coronary ostium were confirmed after the incision of the aorta. Selective cannulation for cardioplegia to the left coronary artery was possible, and cardiac arrest was achieved, but cannulation of the right coronary ostium was impossible. Additional antegrade cardioplegia via SVG was not performed because cardiac arrest was achieved smoothly by initial cardioplegia, and subsequent cardioplegia was performed by retrograde infusion. All aortic valve cups were hypertrophic. The aortic valve was replaced with a 21-mm Carpentier-Edwards PERIMOUNT Magna Aortic Heart Valve (Edwards Lifesciences, Irvine, CA, USA), and the remaining CABG was performed on the LCx with the SVG, to the left descending artery with the LITA, and two proximal anastomoses of the SVG were created at the distal Asc-Ao. The weaning of CPB was possible with supporting IABP. Extubation and the removal of IABP were performed on postoperative day 2. With regard to the antibiotic treatment, the usual perioperative regimen (cefazolin 2 g/day) began 2 days after the surgery and was then converted to syphilis treatment; intravenous administration of penicillin G, 2.4 million units/day (benzathine penicillin is not available in Japan) for 2 weeks and subsequent oral amoxicillin, 750 mg/day. Pathological examination of the aortic wall showed the infiltration of inflammatory cells composed of mainly neutrophiles and lymphocytes localized in the tunica media (Fig. 3a). Concerning the aortic valve, myxoid changes, fibrosis, and the partial infiltration of inflammatory cells were compatible with syphilitic mesaortitis (Fig. 3b). The same pathological changes were detected on the annulus edge of the aortic valve specimen. This patient was transferred to another hospital on postoperative day 25 without any complications.

### Discussion

CVS is classified as the tertiary stage of syphilis infection, and it occurs 20–30 years after the initial infection in about 10% of untreated patients [2]. CVS is associated with aortitis, aortic insufficiency, coronary ostial stenosis, and aortic aneurysm, and patients are generally asymptomatic [5]. In CVS, 14% of patients are reported to have coronary ostial stenosis with AR [6], and about 87% of patients with coronary ostial stenosis have AR [7]. However, bilateral coronary ostial stenosis is rare [7]. Furthermore, AMI due to CVS complicated by both aortic regurgitation and bilateral coronary ostial stenosis, like our case, has been reported only once in English literature [8].

The pathological characteristics of CVS are endarteritis obliterans of the vasa vasorum with chronic inflammatory infiltration, ischemic necrosis, and fibrosis of the tunica media, and this is different from atherosclerotic findings [4]. The pathological results of our case were compatible with these findings and determined to be CVS. Since the preoperative RPR and TPHA tests were positive, antibiotics were administered, but we did not recognize the CVS until the pathological results were reported, as has been previously reported [9, 10]. Cheng [10] has stated, “syphilitic aortitis is dying but not dead,” and Roberts et al. [9] have stated, “cardiovascular syphilis has not disappeared” as well. Additionally, CVS has various characteristic features such as aneurysms and stenotic changes. In our case, computed tomography (CT) was not performed because his hemodynamics were unstable and we did not strongly suspect CVS. If CVS was suspected, CT may be performed before surgery to rule out aortic aneurysms. However, any obvious aortic aneurysms were not detected in the operative field or intraoperative TEE.

Regarding the treatment of coronary ostial lesions, direct reconstructive ostial surgeries such as endarterectomy [11] and vein patch angioplasty [12] have been reported, but not recommended [5], and CABG has been the main treatment in recent years. Considering the histological characteristics of the aortic wall, there are concerns about proximal anastomotic stenosis of the SVG [5]. This risk may decrease by using the internal thoracic artery in situ, but stenosis of the aortic branch is not completely resolved because the syphilitic process has been reported to extend to the aortic arch at a rate of 91% [13] and sometimes to the arch vessels [14]. In our case, a single internal thoracic artery and two saphenous vein grafts were used because of unstable hemodynamics, emergency surgery, and difficulties associated with the administration route for cardioplegia. If possible, the usage of an in situ arterial graft is recommended. To resolve these aortic root problems, root replacement is thought as an alternative option. The guidelines of the American College of Cardiology/American Heart Association [15] have recommended the operative repair of the aortic root or ascending aorta at the class IIa level when the diameter is greater than 4.5–5.0 cm, with some risk factors such as a bicuspid valve and Marfan syndrome. However, this was not performed in our case because the diameters of the aortic sinus and ascending aorta were not greater than 4.5 cm in operative findings, and a short aortic clamping time was required. If the conditions of the guidelines and patients are satisfied, root replacement needs to be strongly considered.

Finally, RPR and TPHA tests of this patient before hospital transfer did not achieve negative conversion. Careful treatment is mandatory for an extended period of time to monitor the recurrence of syphilis, ischemic heart disease due to proximal anastomotic stenosis of the SVG, and other cardiovascular diseases.

## Conclusions

We observed a case of AMI due to CVS with AR and bilateral coronary ostial stenosis requiring CABG and aortic valve replacement. Since CVS does not tend to be recognized in spite of it being a global problem, CVS diagnosis is doubtful depending on blood or imaging examinations, and appropriate treatments including preoperative antibiotic administration should be performed.

## Abbreviations

AMI: Acute myocardial infarction
AR: Aortic regurgitation
Asc-Ao: Ascending aorta
CABG: Coronary artery bypass grafting
CPB: Cardiopulmonary bypass
CT: Computed tomography
CVS: Cardiovascular syphilis
IABP: Intra-aortic bloom pumping
LCx: Left circumflex artery
LITA: Left internal thoracic artery
LV: Left ventricle
RPR: Rapid plasma reagin
SVG: Saphenous vein graft
TEE: Transesophageal echocardiography
TPHA: *Treponema pallidum* hemagglutination
TTE: Transthoracic echocardiography

## References

[1] Hook EW, Peeling RW (2004). Syphilis control—a continuing challenge. N Engl J Med.

[2] Golden MR, Marra CM, Holmes KK (2003). Update on syphilis: resurgence of an old problem. JAMA.

[3] Sekine Y, Yamamoto S, Fujikawa T, Oshima S, Ono M, Sasaguri S (2015). Surgical repair for giant ascending aortic aneurysm to superior vena cava fistula with positive syphilitic test. Gen Thorac Cardiovasc Surg.

[4] Feier H, Cioata D, Teodorescu-Branzeu D, Gaspar M (2012). Coronary ostial stenosis in a young patient. Circulation.

[5] Matsuyama K, Kuinose M, Iida Y, Iwahashi T, Sato K, Iwasaki T (2012). Bilateral coronary ostial stenosis and aortic regurgitation in a patient with cardiovascular syphilis. J Cardiol Cases.

[6] Heggtveit HA (1964). Syphilitic aortitis. A clinicopathologic autopsy study of 100 cases, 1950 to 1960. Circulation.

[7] Bruenn HG (1934). Syphilitic disease of the coronary arteries. Am Heart J.

[8] Choon Ta N, Chee Tang C, Chi KC (2013). Syphilitic coronary artery ostial stenosis presenting with acute myocardial infarction. Heart.

[9] Roberts WC, Bose R, Ko JIM, Henry AC, Hamman BL (2009). Identifying cardiovascular syphilis at operation. Am J Cardiol.

[10] Cheng TO (2001). Syphilitic aortitis is dying but not dead. Cathet Cardiovasc Intervent.

[11] Dubost C, Blondeau P, Piwnica A, Weiss M, Lenfant C, Passelecq J (1960). Syphilitic coronary obstruction: correction under artificial heart-lung and profound hypothermia at 10°C. Surgery.

[12] Croti UA, Gregori F, Marcial MB, Dallan LA, Gregori TEF, Oliveira DS (2000). Coronary bilateral ostial enlargement using the saphenous vein in a patient with syphilitic. Arq Bras Cardiol.

[13] Roberts WC, Ko JM, Vowels TJ (2009). Natural history of syphilitic aortitis. Am J Cardiol.

[14] Jackman JD, Radolf JD (1989). Cardiovascular syphilis. Am J Med.

[15] Nishimura RA, Otto CM, Bonow RO (2014). 2014 AHA/ACC guideline for the management of patients with valvular heart disease: executive summary: a report of the American College of Cardiology/American Heart Association Task Force on Practice Guidelines. Circulation.

